# Supplementary material for: Nanoscale cellular organization of viral RNA and proteins in SARS-CoV-2 replication organelles
Source: Nat Commun. 2024 May 31;15:4644. doi: 10.1038/s41467-024-48991-x (PMC11143195; doi:10.1038/s41467-024-48991-x)
Supplement: Supplementary file 1 — Supplementary Information [file 41467_2024_48991_MOESM1_ESM.pdf]

Supplementary figures

Nanoscale cellular organization of viral RNA and proteins in SARS-CoV-2 replication organelles

Leonid Andronov*[1], Mengting Han*[2], Yanyu Zhu[2], Ashwin Balaji[1,3], Anish R. Roy[1], Andrew E. S. Barentine[1], Puja Patel[4], Jaishree Garhyan[4], Lei S. Qi[2,5,6,#], W.E. Moerner[1,5,#]

[1]Department of Chemistry; [2]Department of Bioengineering; [3]Biophysics PhD Program; Stanford University, Stanford, CA 94305 U.S.A.

[4]In Vitro Biosafety Level 3 (BSL-3) Service Center, School of Medicine; Stanford University, Stanford, CA 94305 U.S.A.

[5]Sarafan ChEM-H; Stanford University, Stanford, CA 94305 U.S.A.

[6]Chan Zuckerberg Biohub – San Francisco, San Francisco, CA 94158 U.S.A.

*These authors contributed equally.

#Correspondence to: W. E. Moerner, wmoerner@stanford.edu; Lei S. Qi, sqi@stanford.edu

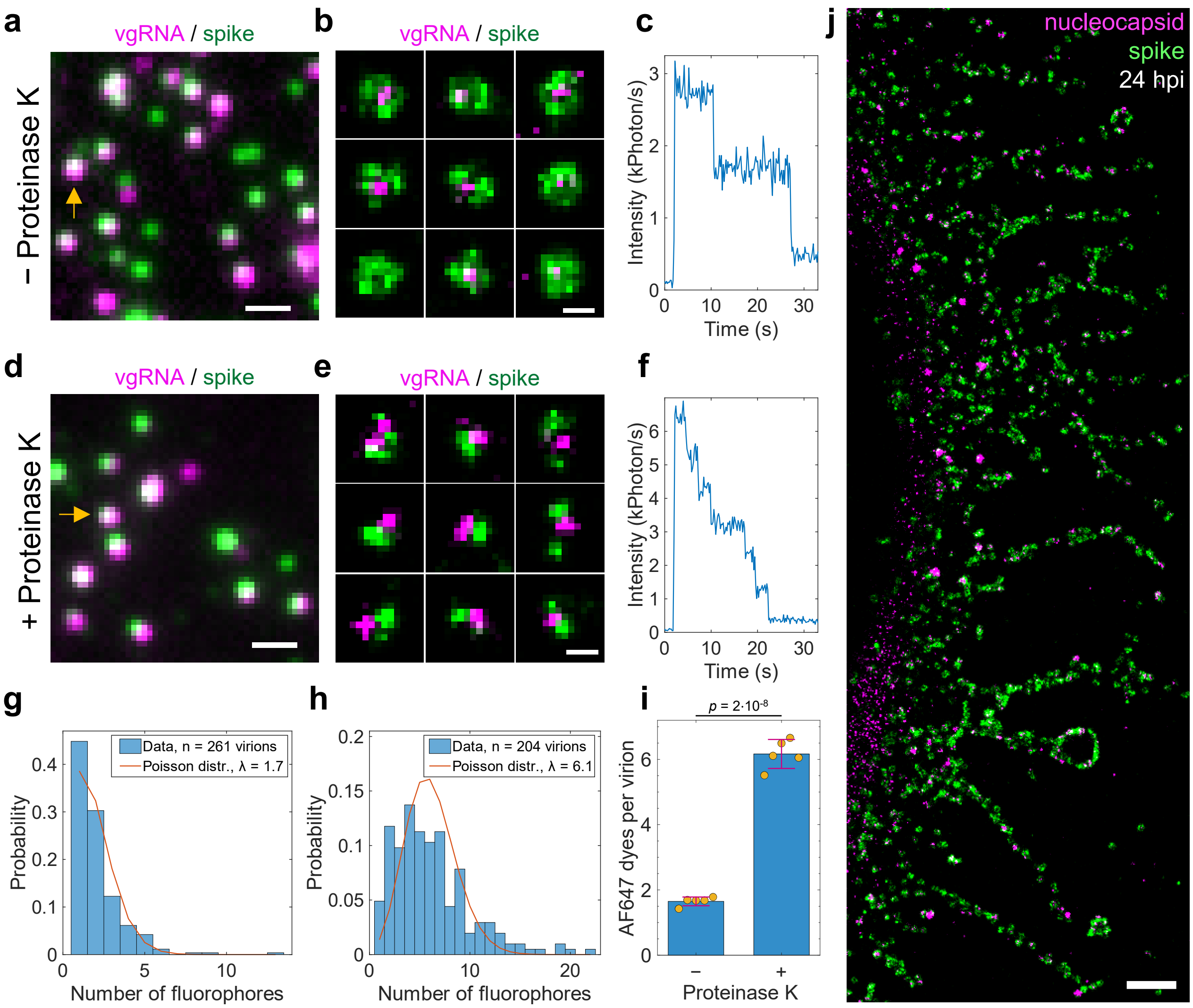

Supplementary Fig. S1. Validation of the labeling and imaging approach.

a, DL image of SARS-CoV-2 virions where vgRNA was labeled with AF647 by RNA FISH and the spike proteins were labeled by primary anti-spike S2 antibody with secondary CF568-conjugated antibody. **b**, Representative two-color SR images of individual virions reveal concentric localization of spike around vgRNA. **c**, Bleaching time trace of AF647 emission from a single virion (yellow arrow in **a**) demonstrates two-step bleaching. **d**, DL image of virions that were treated with Proteinase K (PK) before labeling. **e**, SR images of PK-treated virions reveal incomplete spike labeling due to digestion of proteins by the PK. **f**, Bleaching time trace of AF647 emission from a single virion (yellow arrow in **d**) shows 6-step bleaching suggesting increased vgRNA labeling efficiency in PK-treated virions. **g**-**h**, Histograms of the number of fluorophores per virion in untreated (**g**) or PK-treated (**h**) samples and their fits with a Poisson distribution. **i**, Mean number of AF647 molecules per virion from the fit for 5 different regions in both untreated and PK-treated samples. The error bars indicate mean ± SD values for the untreated and PK-treated groups. **j**, SR image of a SARS-CoV-2 infected cell with the cell body to the left reveals assembled virions at its cytoplasmic tubular projections at 24 hpi. Scale bars, 100 nm (**b**, **e**) and 1 μm (**a**, **d**, **j**).

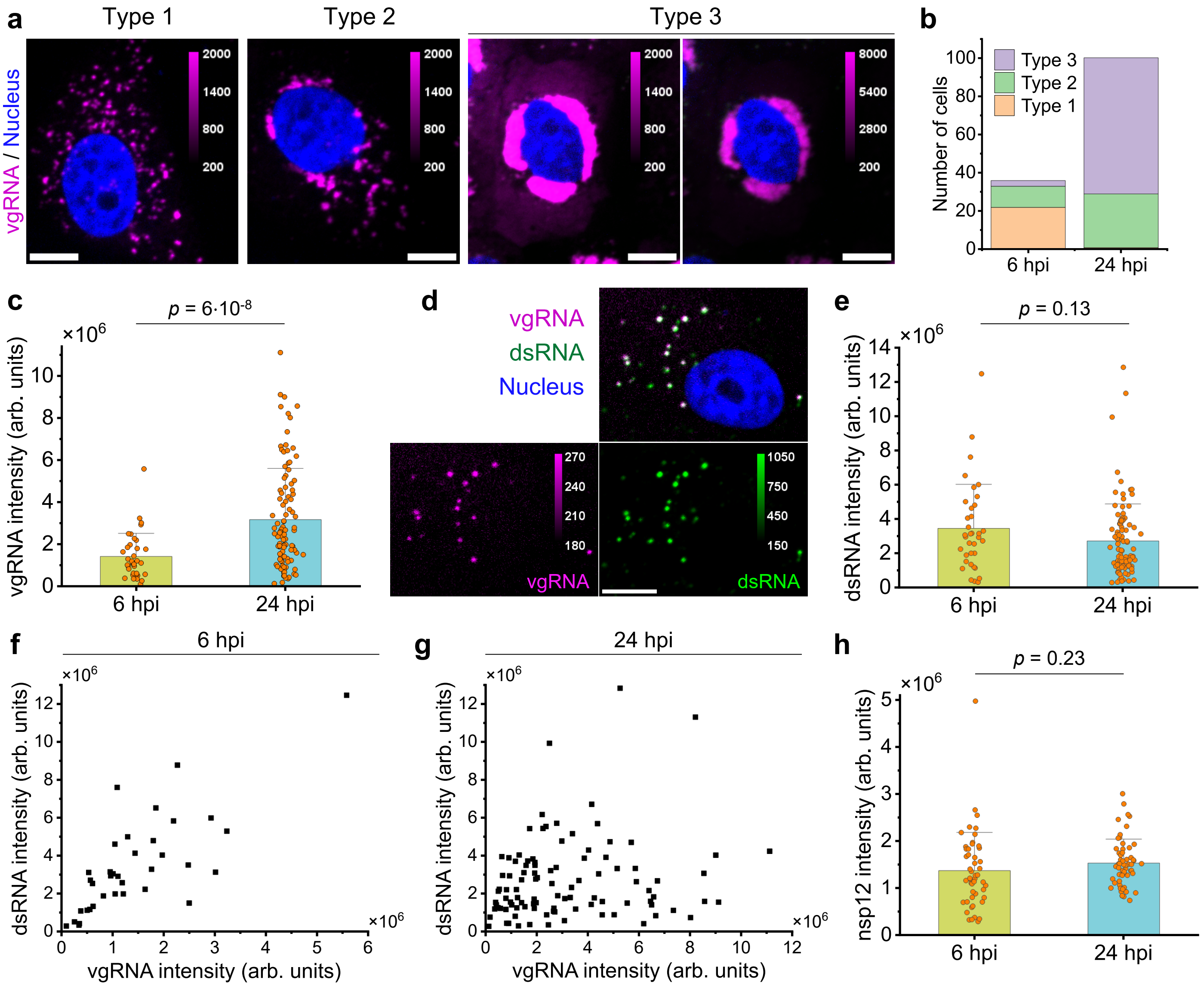

Supplementary Fig. S2. Screening and quantification of vgRNA, dsRNA and nsp12 by confocal microscopy.

a, Representative confocal images show three types of vgRNA distribution in SARS-CoV-2 infected cells. **b**, Number of cells assigned to one of the three types at 6 or 24 hpi. **c**, Cell-integrated vgRNA signal significantly increases from 6 hpi to 24 hpi. **d**, Representative confocal image of vgRNA and dsRNA in an early type 1 cell suggests colocalization between these targets. **e**, Cell-integrated signal of immunofluorescently detected dsRNA in SARS-CoV-2 infected cells does not significantly change from 6 hpi to 24 hpi. **f**, dsRNA signal correlates with vgRNA signal at 6 hpi (Pearson's r = 0.76). **g**, dsRNA signal does not correlate with vgRNA signal at 24 hpi (Pearson's r = 0.18). **h**, Cell-integrated signal of immunofluorescently detected nsp12 in SARS-CoV-2 infected cells does not significantly change from 6 hpi to 24 hpi. Error bars represent mean + SD of the values from individual cells. Scale bars, 10 µm.

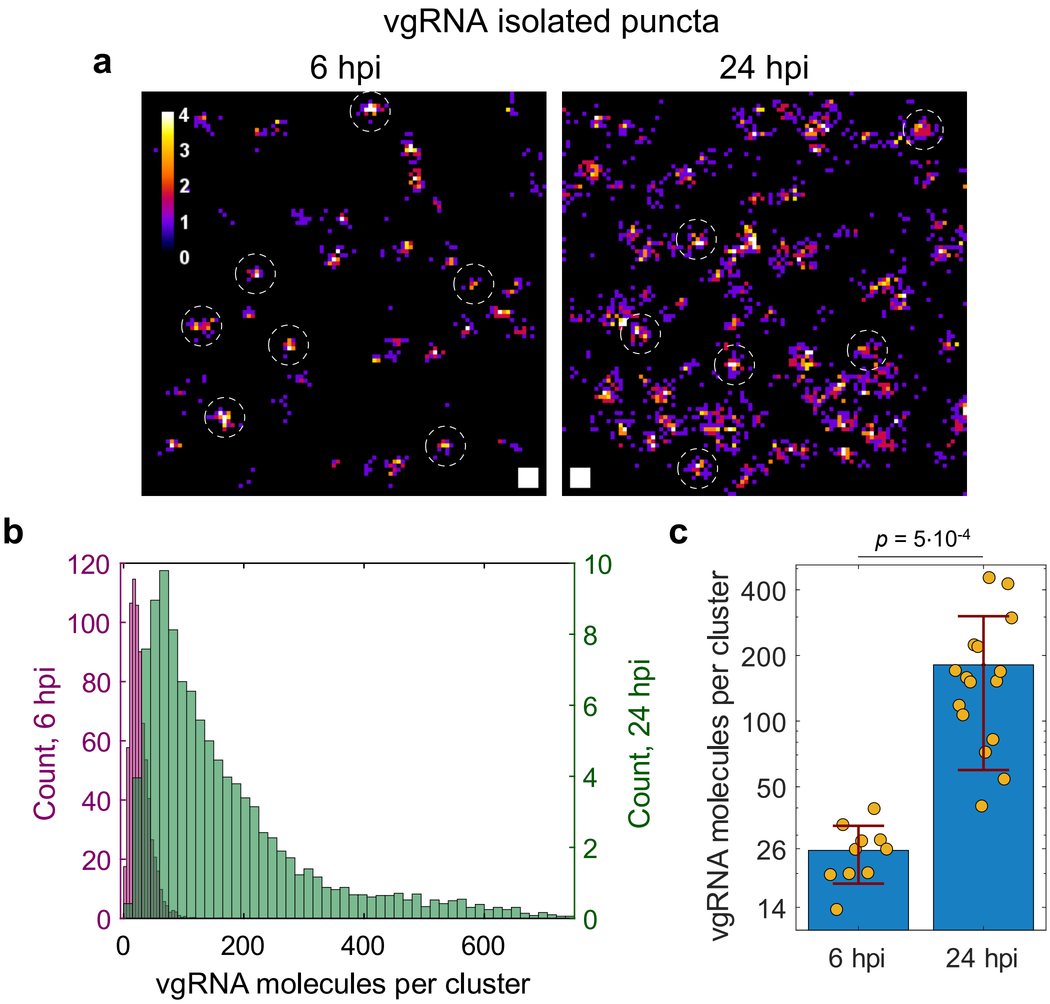

Supplementary Fig. S3. Estimation of the number of vgRNA molecules in vgRNA clusters.

a, SR localizations of single vgRNA molecules found in the cytoplasm of infected cells outside the dense vgRNA clusters. On a cell-by-cell basis, similar images are used as a calibration for the number of SR detections per one vgRNA molecule. Examples of SR images of single vgRNA molecules are indicated with white circles (r = 50 nm). **b**, Estimated number of vgRNA molecules per cluster at 6 and 24 hpi from all analyzed cells. The histogram counts are normalized by the number of analyzed cells; the histogram counts for 24 hpi were additionally divided by 3 to account for the 3× wider bin size than at 6 hpi. **c**, Median estimated counts of vgRNA molecules per cluster for each analyzed cell (individual yellow points). The error bars represent mean ± SD values of these median counts for each time point. For data in **b** and **c**, 10 cells were analyzed for 6 hpi and 16 cells for 24 hpi, using 2 independent experiments for each time point. Scale bars, 50×50 nm^2.

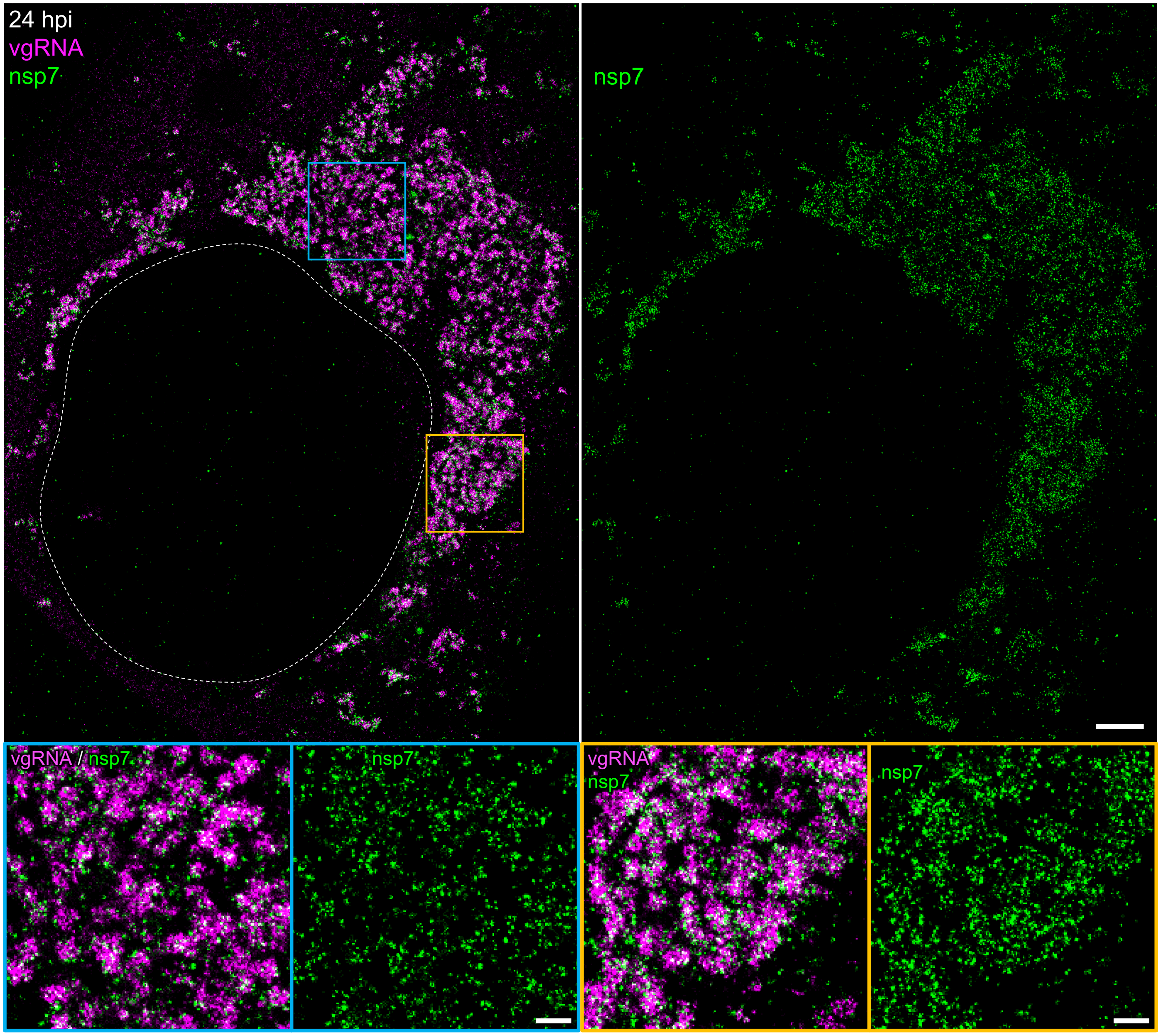

Supplementary Fig. S4. Association of nsp7 with perinuclear vgRNA structures.

Representative SR image of a SARS-CoV-2 infected cell at 24 hpi labeled for vgRNA (magenta) and nsp7 (green) with magnified regions shown in the colored boxes. Scale bars, 2 µm and 500 nm (bottom panels).

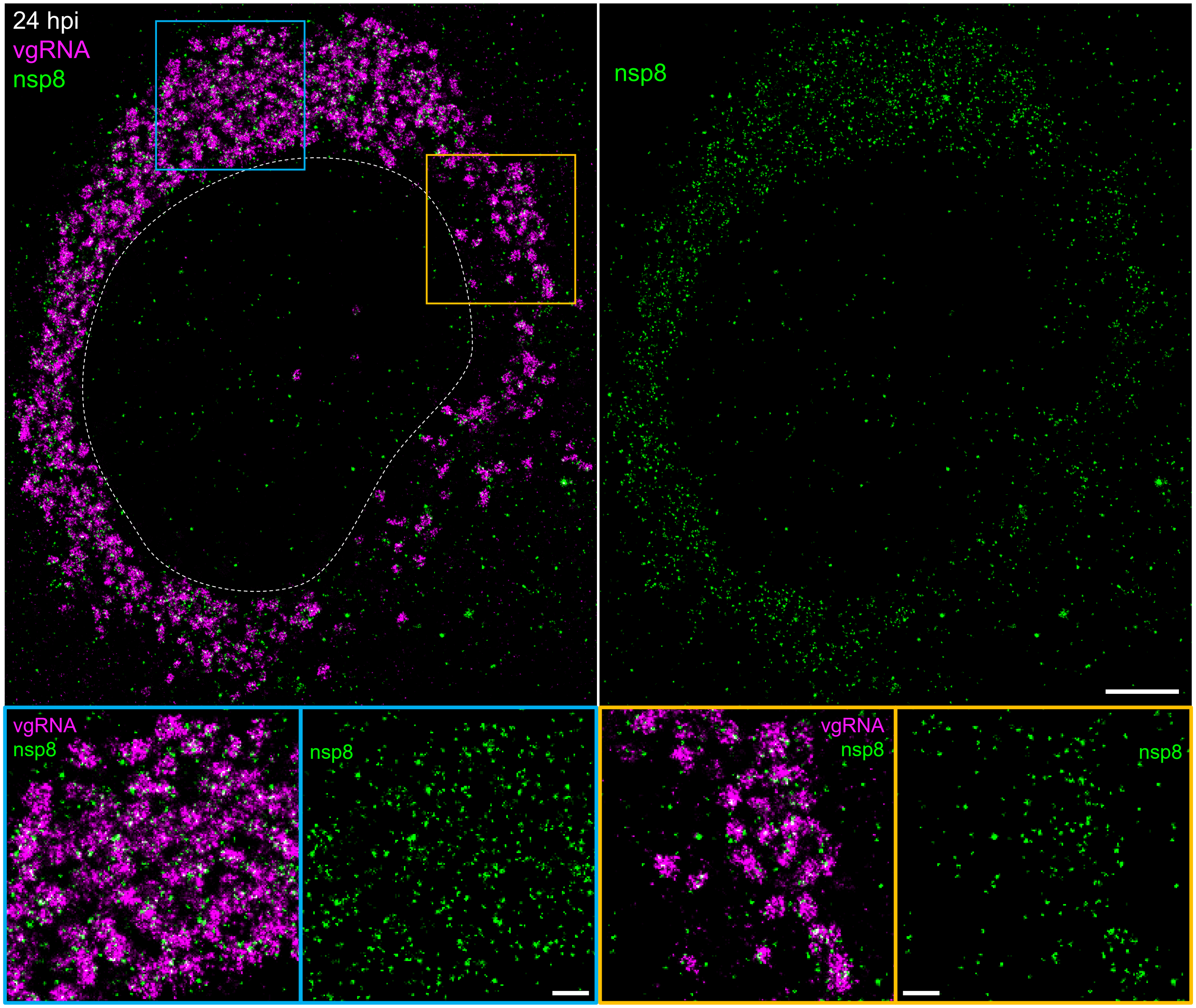

Supplementary Fig. S5. Association of nsp8 with perinuclear vgRNA structures.

Representative SR image of a SARS-CoV-2 infected cell at 24 hpi labeled for vgRNA (magenta) and nsp8 (green) with magnified regions shown in the colored boxes. Scale bars, 2 µm and 500 nm (bottom panels).

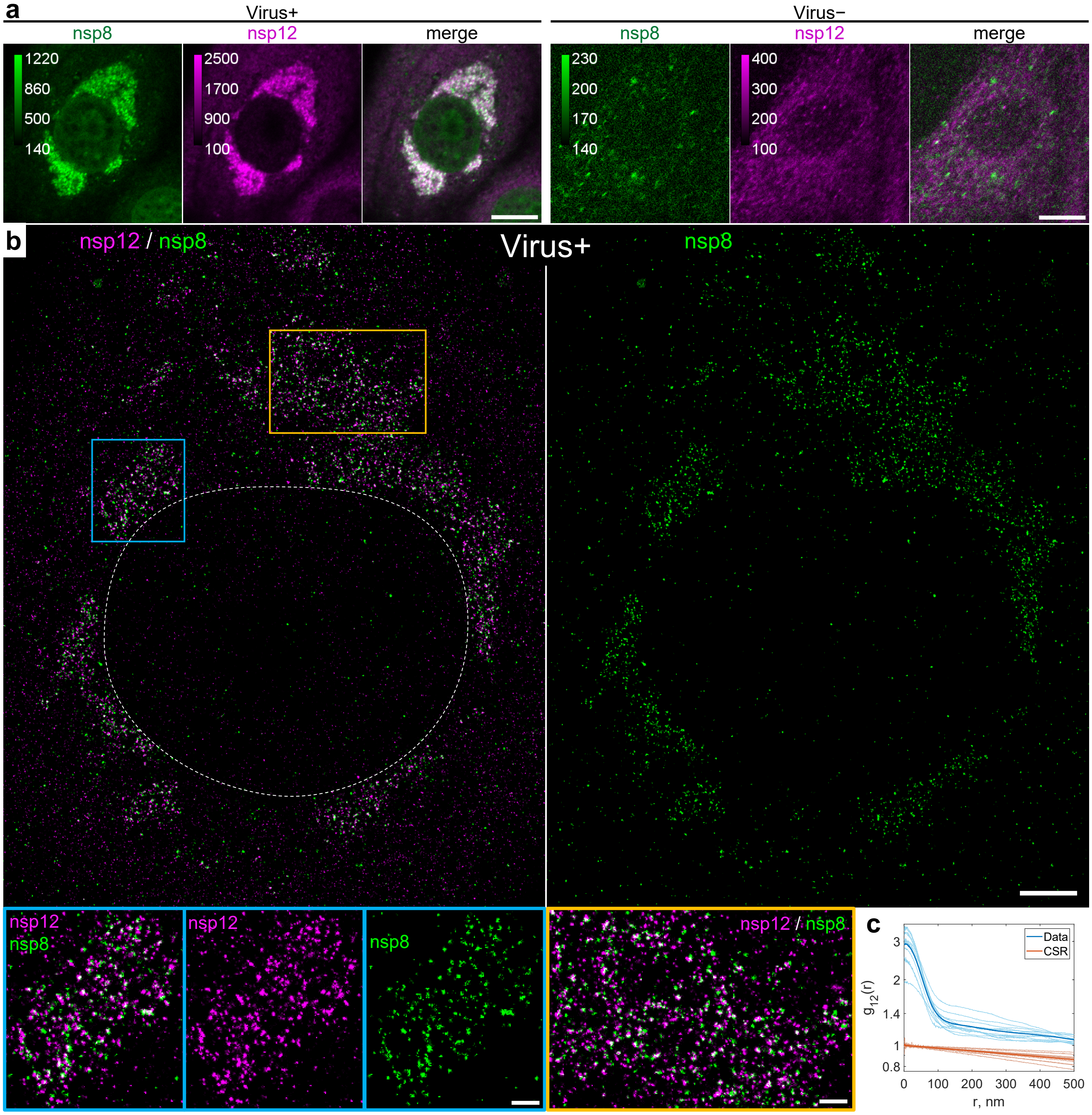

Supplementary Fig. S6. Colocalization of nsp12 with nsp8.

a, Representative confocal images of cells co-labeled for nsp8 and nsp12 demonstrate their DL colocalization in the perinuclear region of infected cells (Virus+, 24 hpi) and low background immunofluorescence signal in non-infected cells (Virus−). **b**. Representative SR image of an infected cell at 24 hpi reveals punctate localization of both nsp12 and nsp8 in the perinuclear region. (bottom panels) Magnified images of the regions in the colored boxes reveal nanoscale colocalization of nsp12 with nsp8. **c**. Bivariate pair-correlation functions calculated in the perinuclear regions of infected cells demonstrate colocalization of nsp12 and nsp8 at $r < 100$ nm. Scale bars, 10 µm (**a**), 2 µm (**b**) and 500 nm (bottom panels).

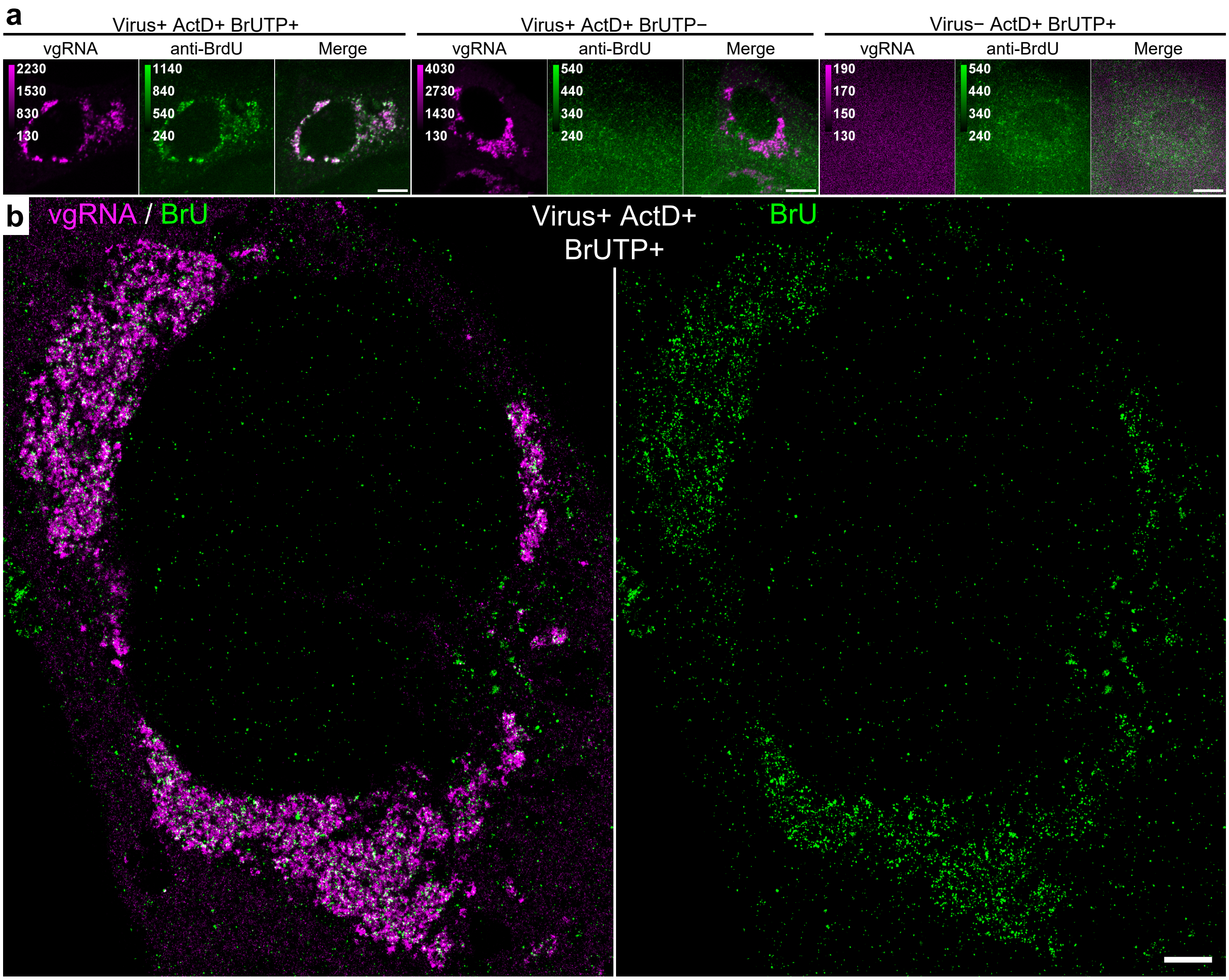

Supplementary Fig. S7. Association of newly synthesized viral RNAs with perinuclear clusters of vgRNA.

a, Representative confocal images of cells co-labeled for vgRNA and BrU demonstrate their DL colocalization in the perinuclear region of infected cells treated with BrUTP for 1 h before fixation (Virus+ BrUTP+); low background BrU signal in infected cells not treated with BrUTP (Virus+ BrUTP−) and low background signal of both targets in non-infected cells treated with BrUTP for 1 h (Virus− BrUTP+). Endogenous transcription was inhibited with Actinomycin D in all conditions (ActD+). Virus+ cells were fixed at 24 hpi. **b**. Representative SR image of an infected cell at 24 hpi treated with BrUTP and Actinomycin D demonstrates association of BrU labeling with vgRNA clusters. Scale bars, 10 µm (**a**), 2 µm (**b**).

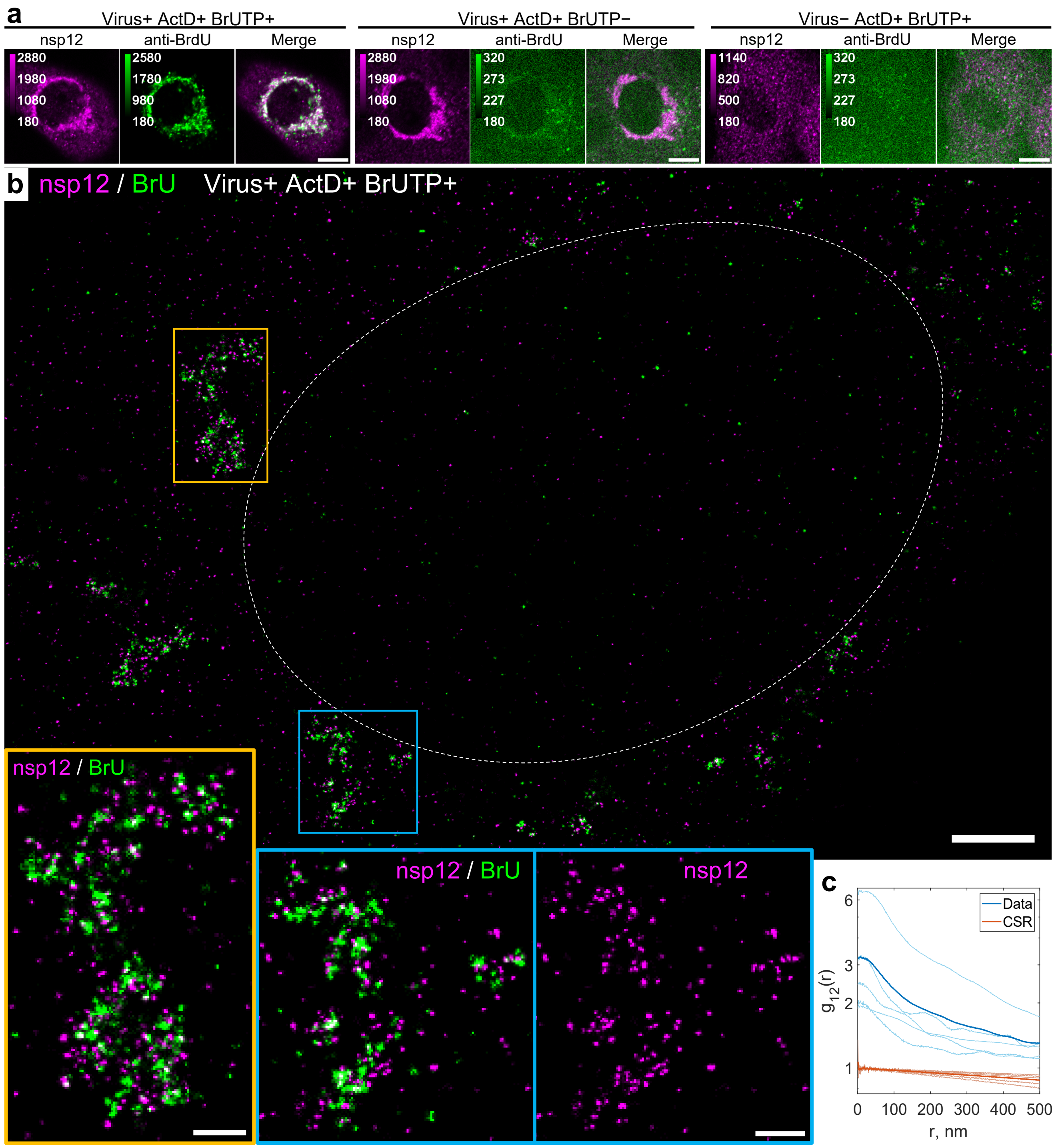

Supplementary Fig. S8. Association of newly synthesized viral RNAs with nsp12.

a, Representative confocal images of cells co-labeled for nsp12 and BrU demonstrate their DL colocalization in the perinuclear region of infected cells treated with BrUTP for 1 h (Virus+ BrUTP+); low background BrU signal in infected cells not treated with BrUTP (Virus+ BrUTP−); and low background signal of both targets in non-infected cells treated with BrUTP for 1 h (Virus− BrUTP+). Endogenous transcription was inhibited with Actinomycin D in all conditions (ActD+). Virus+ cells were fixed at 24 hpi. **b**. SR image of an infected cell (type 1, early infection) treated with BrUTP demonstrates association of BrU labeling with nsp12. **c**. Bivariate pair-correlation functions calculated in the perinuclear regions of infected and BrUTP-treated cells reveal nanoscale association of nsp12 and BrU. Scale bars, 10 µm (**a**), 2 µm (**b**) and 500 nm (bottom zoomed-in panels).

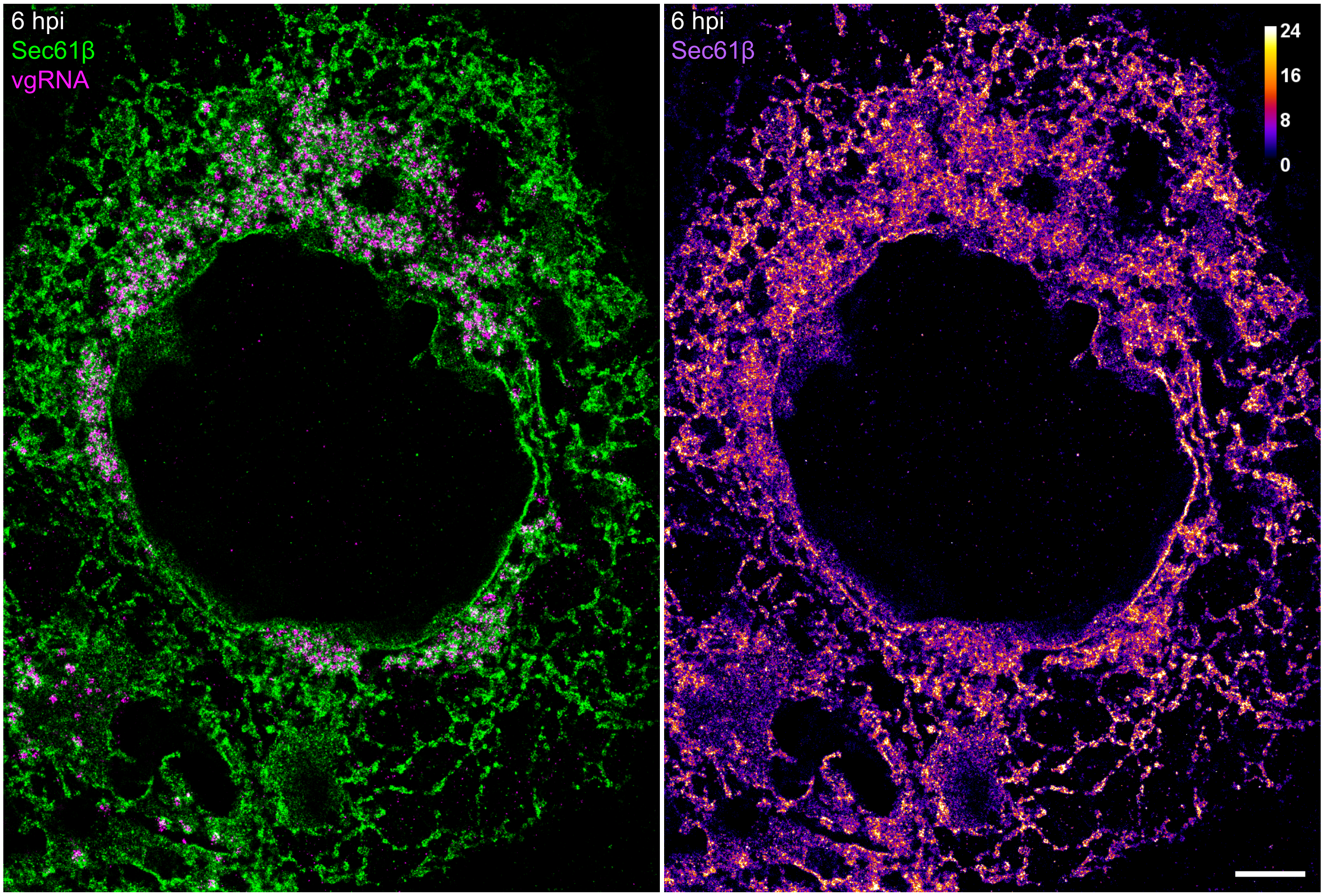

Supplementary Fig. S9. Alterations of host cell ER at 6 hpi.

Representative SR image of vgRNA in a SARS-CoV-2 infected Vero E6 cell, stably expressing Sec61β-GFP. Altered ER forms ring-like structures that partially encapsulate vgRNA clusters in the perinuclear region. Left: green (Sec61β) / magenta (vgRNA) coloring; right: color scale of Sec61β localizations. Scale bar, 2 µm.

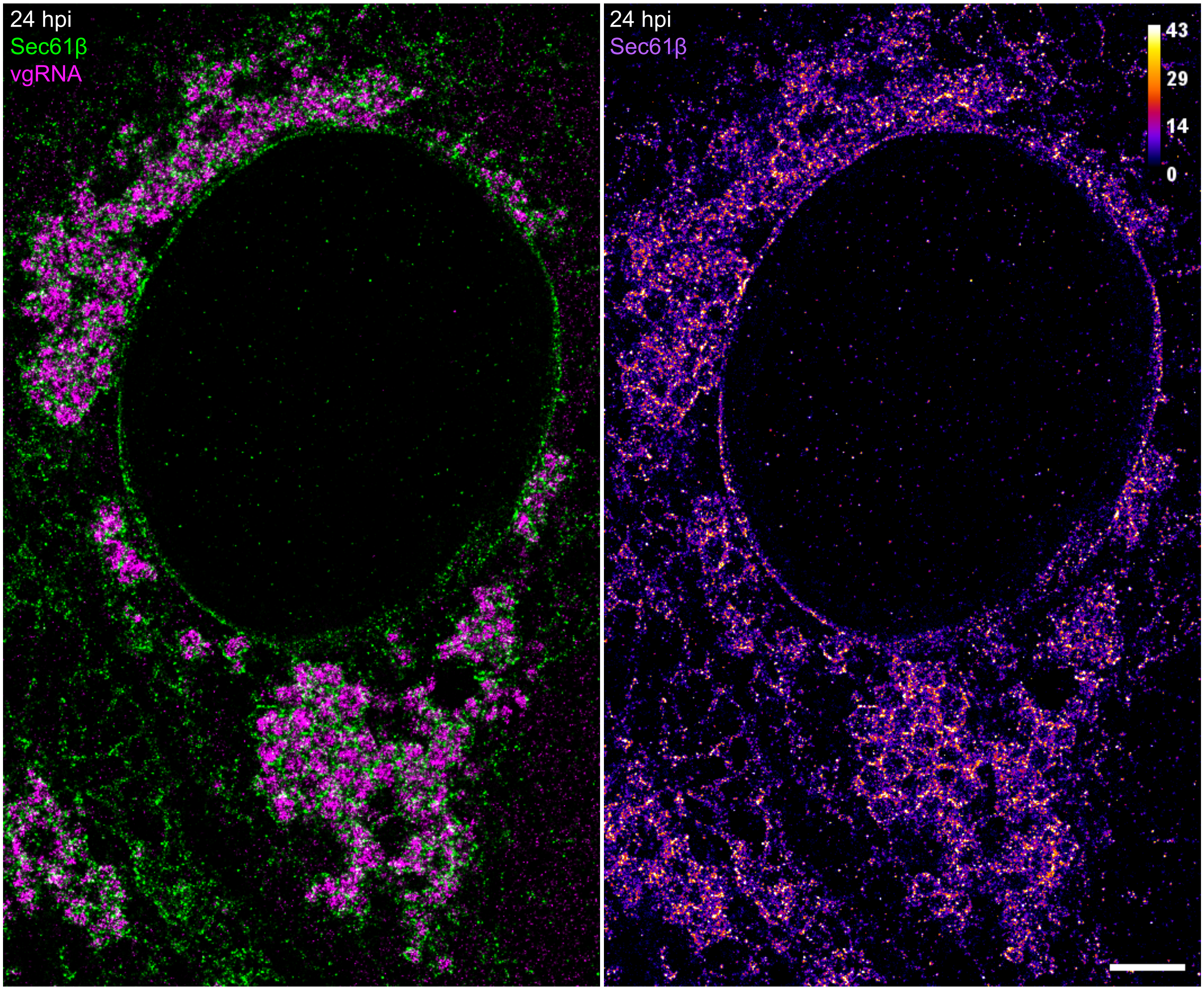

Supplementary Fig. S10. Alterations of host cell ER at 24 hpi.

Representative SR image of vgRNA in a SARS-CoV-2 infected Vero E6 cell, stably expressing Sec61β-GFP. Altered ER forms ring-like structures that encapsulate vgRNA clusters in the perinuclear region, while the Sec61β signal at the ER tubules decreases compared to 6 hpi (Supplementary Fig. S9). Left: green (Sec61β) / magenta (vgRNA) coloring; right: color scale of Sec61β localizations. Scale bar, 2 µm.

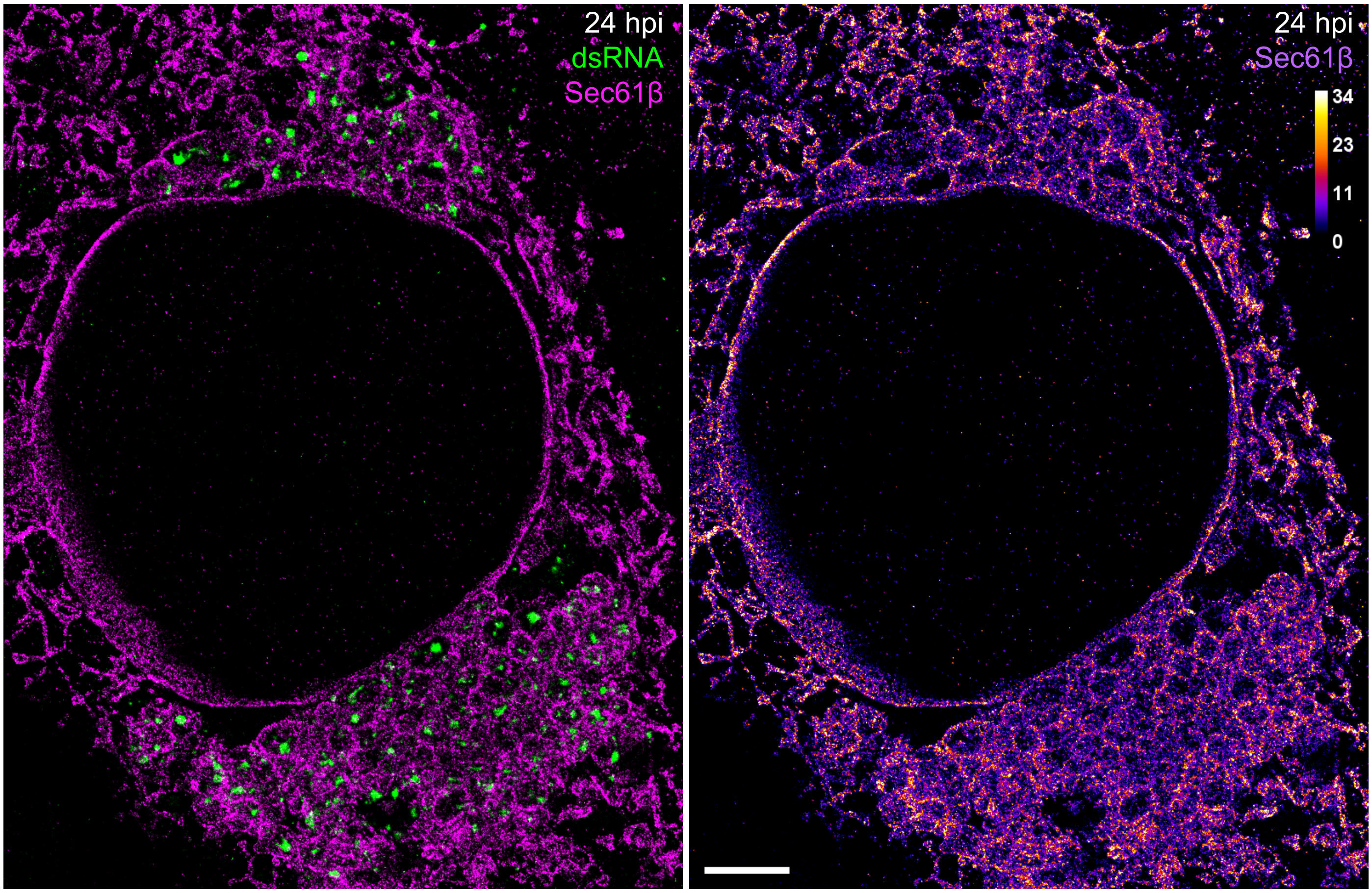

Supplementary Fig. S11. Encapsulation of dsRNA by altered host ER at 24 hpi.

Representative SR image of dsRNA in a SARS-CoV-2 infected Vero E6 cell, stably expressing Sec61β-GFP. Ring-like structures of altered ER encapsulate dsRNA clusters in the perinuclear region. Left: green (dsRNA) / magenta (Sec61β) coloring; right: color scale of Sec61β localizations. Scale bar, 2 µm.

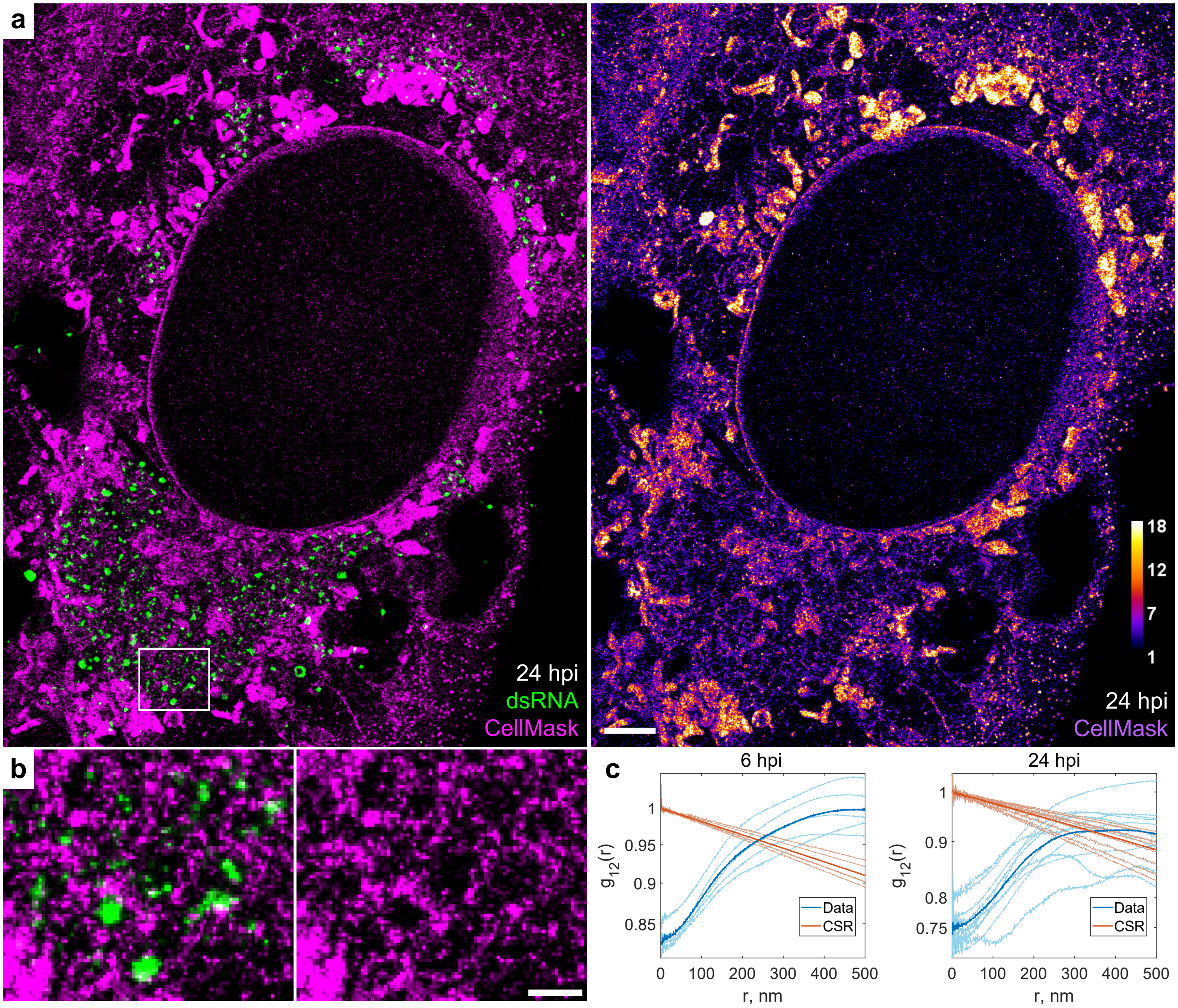

Supplementary Fig. S12. Encapsulation of dsRNA into membrane-bound organelles.

a, Representative SR image of dsRNA and membranes in a SARS-CoV-2 infected cell at 24 hpi with membranes labeled by CellMask Deep Red (magenta) and dsRNA labeled with immunofluorescence (green). CellMask-labeled membranes can be observed around dsRNA clusters. Virions at the plasma membrane are seen as bright puncta (right side and lower right corner of the image). **b**, Zoomed-in image that corresponds to the white box in **a**. **c**, Bivariate pair-correlation functions indicate nanoscale anti-correlation between dsRNA and CellMask, consistent with dsRNA encapsulation in membrane-bound organelles at both 6 and 24 hpi. Scale bars, 2 µm (**a**) and 500 nm (**b**).

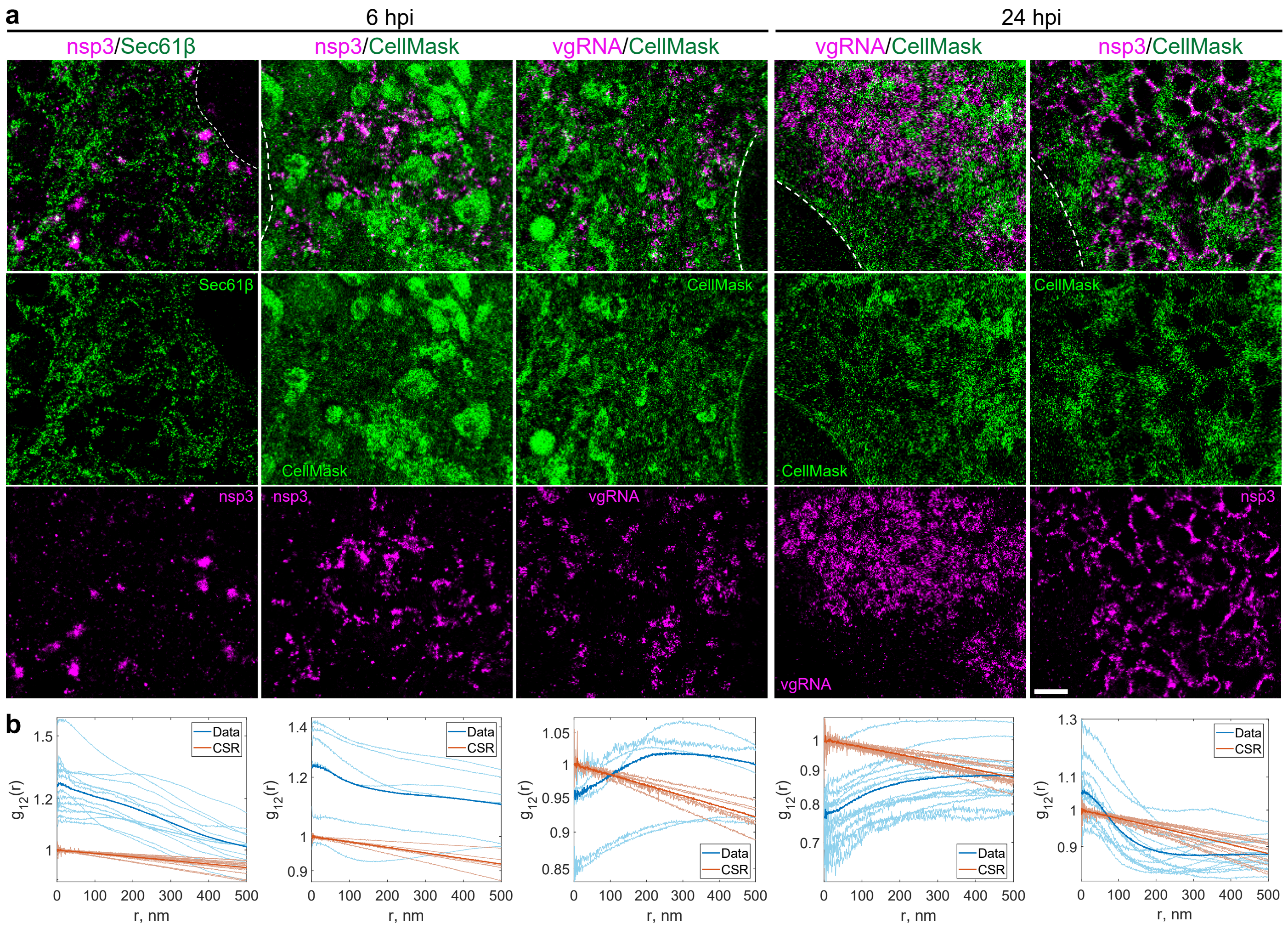

Supplementary Fig. S13. Nanoscale co-organization of viral components with host cell membranes.

a, Representative SR images of nsp3, Sec61β, vgRNA and membranes (CellMask) in SARS-CoV-2 infected cells at 6 and 24 hpi. **b**, Bivariate pair-correlation functions indicate nanoscale association between nsp3 and Sec61β, nsp3 and CellMask, and nanoscale anti-correlation between vgRNA and CellMask at both time points. Scale bar, 1 µm.

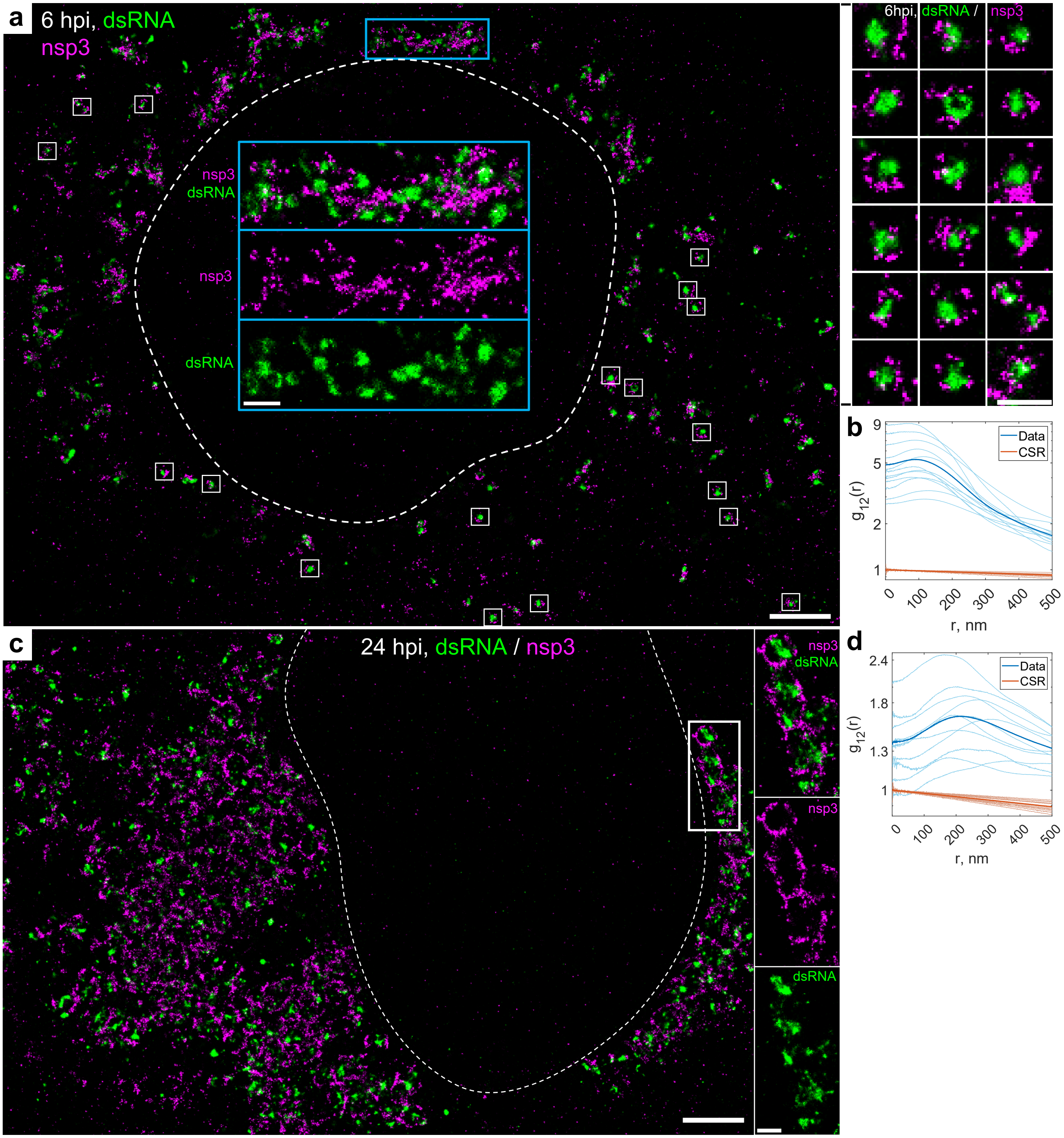

Supplementary Fig. S14. Nanoscale anti-correlation of nsp3 with dsRNA.

a, Representative SR image of a SARS-CoV-2 infected cell at 6 hpi with nsp3 and dsRNA labeled by immunofluorescence. Nsp3 can be observed at the surface of isolated dsRNA clusters (white boxes & right panel) or in dense aggregates between dsRNA clusters (blue box & blue insets). **b**, Bivariate pair-correlation functions indicate nanoscale anti-correlation between dsRNA and nsp3 at 6 hpi. **c**, SR image of a SARS-CoV-2 infected cell at 24 hpi. Nsp3 forms a network-like pattern that encapsulates dsRNA clusters. **d**, Bivariate pair-correlation functions indicate nanoscale anti-correlation between dsRNA and nsp3 at 24 hpi. Scale bars, 2 µm (**a**, **c**) and 500 nm (insets in **a**, **c** and right panel in **a**).

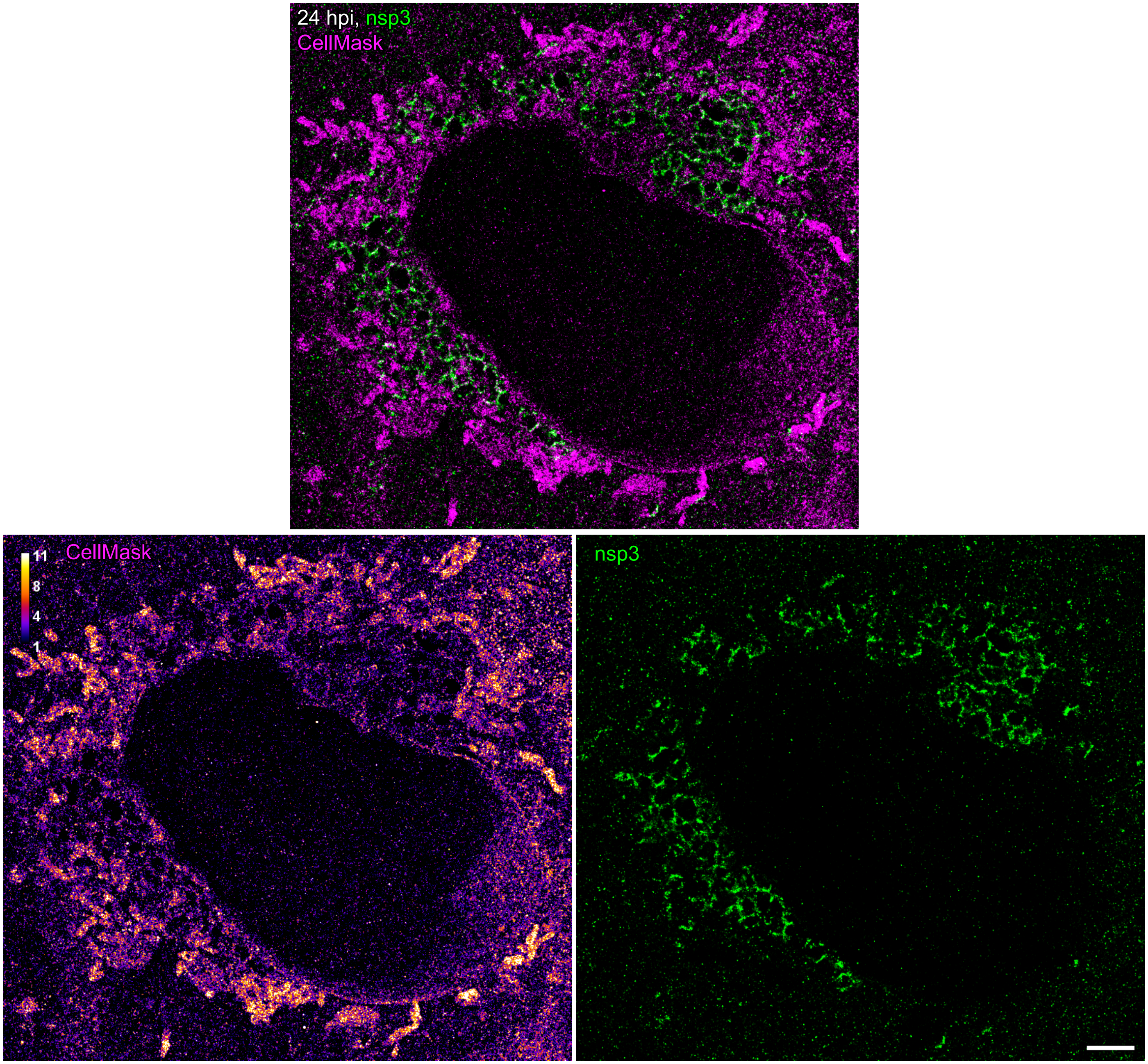

Supplementary Fig. S15. Nanoscale colocalization of nsp3 with membranes at 24 hpi.

Representative SR image of nsp3 (green) and membranes as labeled by CellMask (magenta) in a SARS-CoV-2 infected cell at 24 hpi. Nsp3 forms a network-like pattern in the perinuclear region that colocalizes with the CellMask pattern. Scale bar, 2 µm.

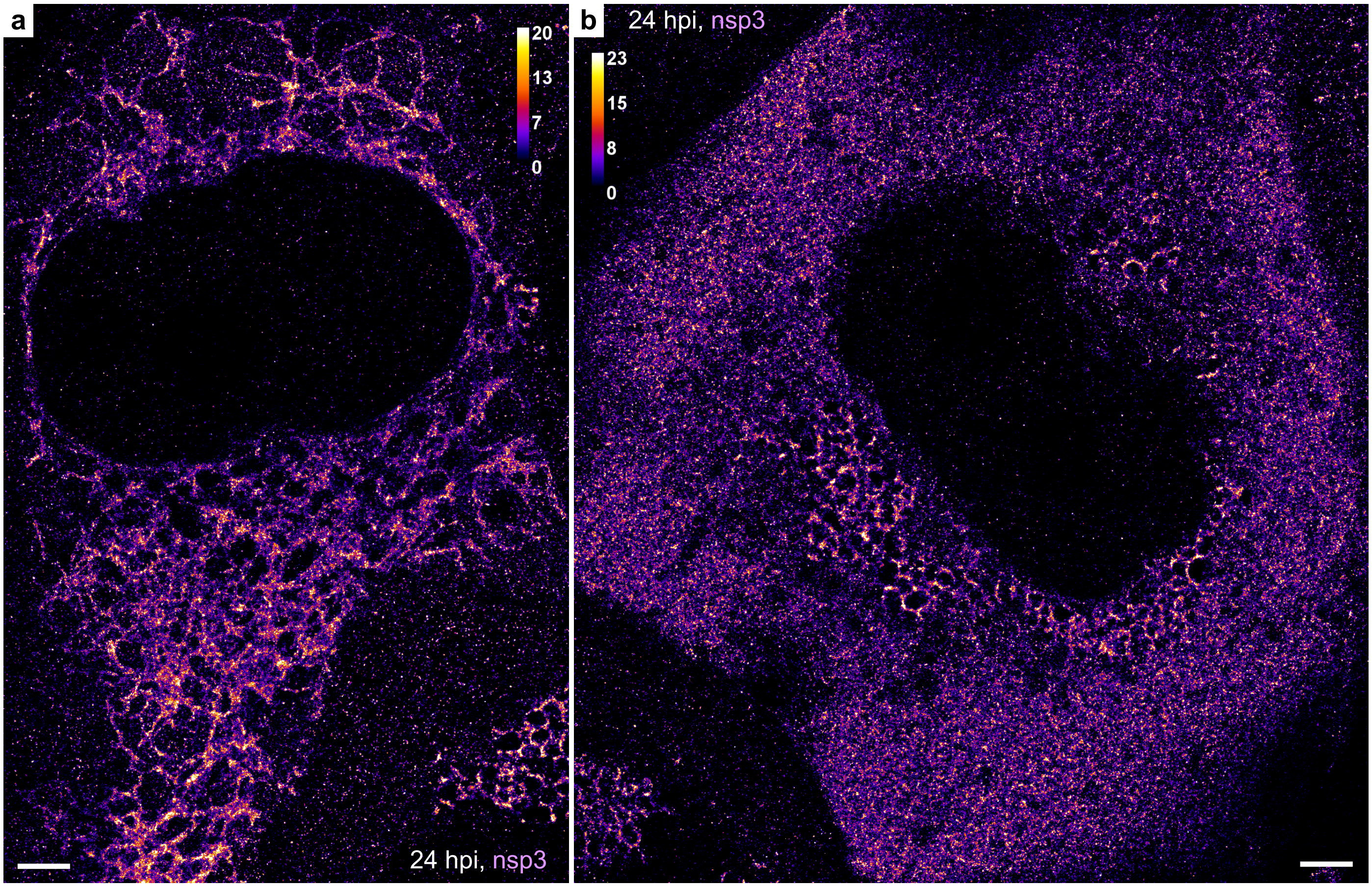

Supplementary Fig. S16. Less common patterns of nanoscale nsp3 localization at 24 hpi.

a, Nsp3 forms an ER-like network that occupies a large part of the cytoplasm. **b**, Besides the common perinuclear pattern, nsp3 is also diffusely localized throughout the whole cytoplasm. Scale bars, 2 µm.

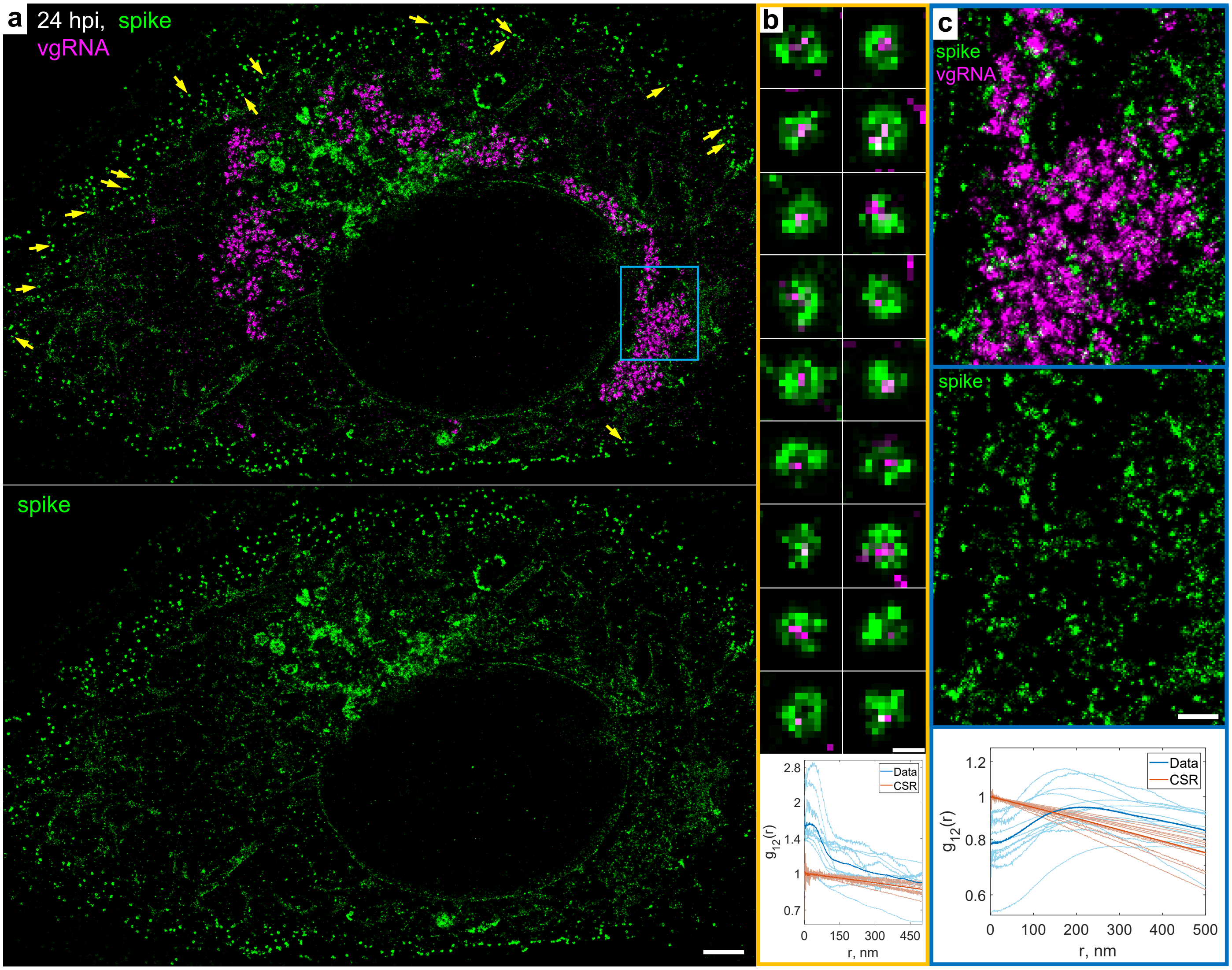

Supplementary Fig. S17. Nanoscale localization of spike protein at 24 hpi.

a, Representative SR image of a SARS-CoV-2 infected cell at 24 hpi labeled for spike (green) and vgRNA (magenta). **b**, Examples of assembled virions encapsulated by the spike proteins and with vgRNA in their interior, detected at the cell periphery (yellow arrows in **a**). (bottom panel) Bivariate pair-correlation functions calculated in the plasma membrane regions indicate colocalization of these targets at $r < 100$ nm. **c**, Magnified image that corresponds to the blue frame in **a** displays spike localizations mostly excluded from the interior of the perinuclear vgRNA clusters with possible localization at their membrane. (bottom panel) Bivariate pair-correlation functions calculated in the perinuclear regions of infected cells indicate nanoscale anti-correlation of spike with SARS-CoV-2 replication organelles. Scale bars, 2 µm (**a**), 100 nm (**b**), 500 nm (**c**).

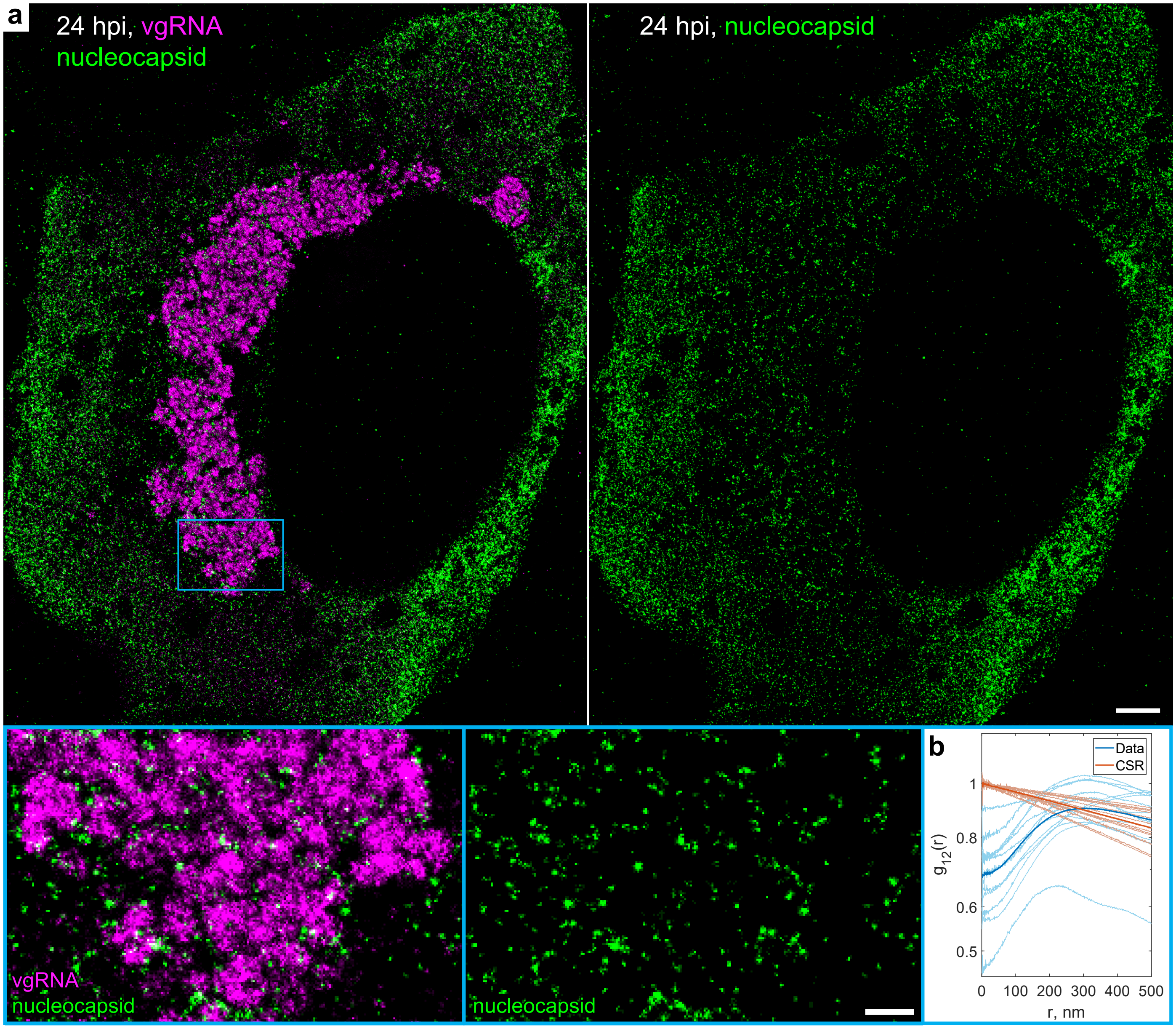

Supplementary Fig. S18. Nanoscale anti-correlation of nucleocapsid protein with SARS-CoV-2 replication organelles at 24 hpi.

a, Representative SR image of a SARS-CoV-2 infected cell at 24 hpi labeled for the nucleocapsid protein (green) and vgRNA (magenta). The magnified image in the blue frame displays nucleocapsid protein localizations mostly excluded from the interior of the perinuclear vgRNA clusters with possible localization at their membrane. **b**, Bivariate pair-correlation functions calculated in the perinuclear regions of the infected cells indicate nanoscale anti-correlation of the nucleocapsid protein with vgRNA. Scale bars, 2 µm and 500 nm (bottom panels).

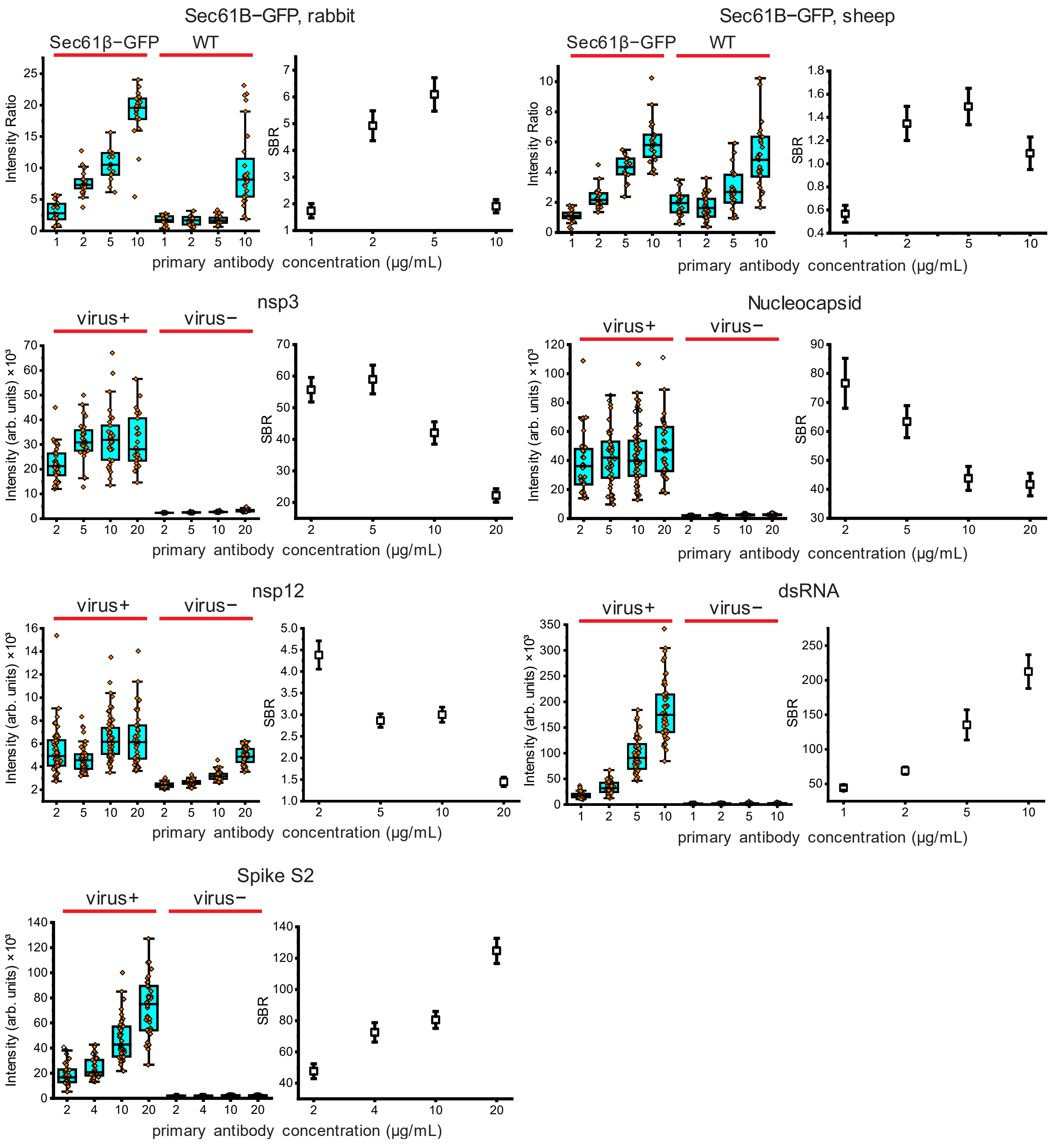

Supplementary Fig. S19. Optimization of primary antibody concentrations.

The concentration of primary antibodies was optimized to minimize the background or to maximize the signal to background ratio (SBR) between SARS-CoV-2 infected and non-infected cells or between cells expressing Sec61β-GFP and WT cells (Methods). Box plots: center line, median; box limits, upper and lower quartiles; whiskers, 1.5× interquartile range; dots, values for individual cells. SBR plots show mean ± SD.

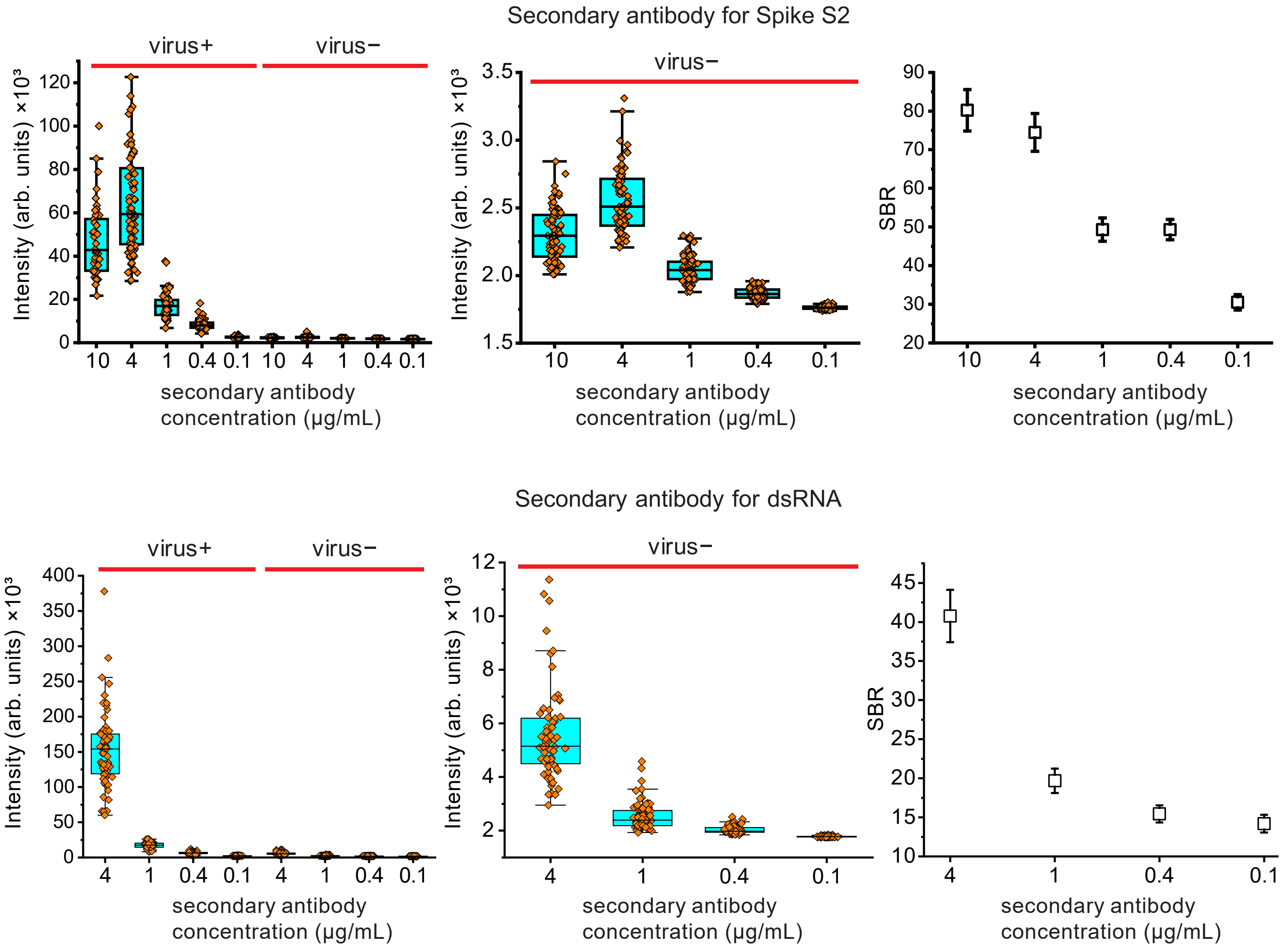

Supplementary Fig. S20. Optimization of secondary antibody concentrations.

The concentration of secondary antibodies was optimized to minimize the background or to maximize the SBR between SARS-CoV-2 infected and non-infected cells (see Methods). Box plots: center line, median; box limits, upper and lower quartiles; whiskers, 1.5× interquartile range; dots, values for individual cells. SBR plots show mean ± SD.

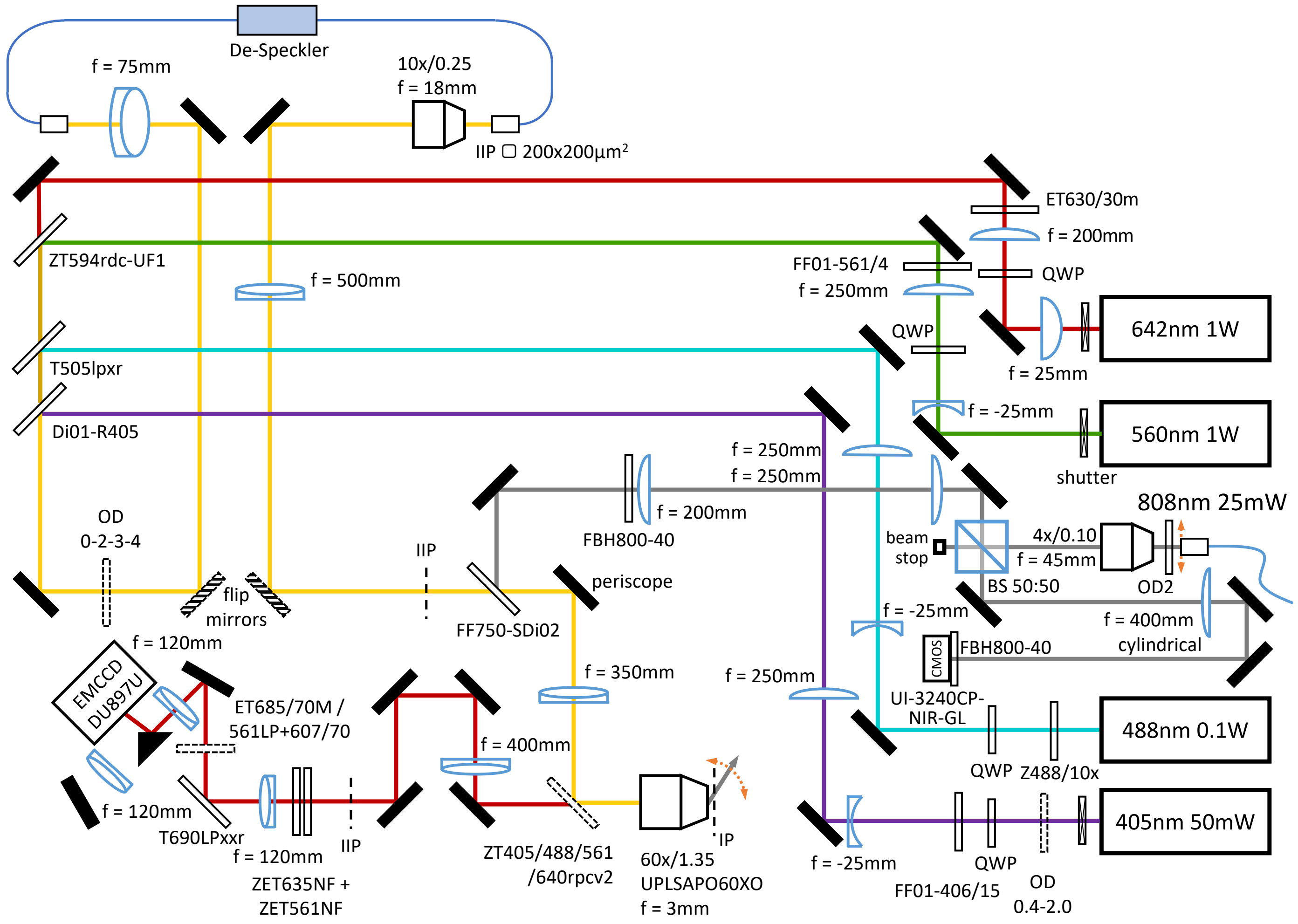

Supplementary Fig. S21. Path diagram of SR microscope used in this study.

Black-filled icons: mirrors; thin empty rectangles: dichroic or neutral density filters; dashed rectangles: movable or motorized components; boxes: cameras or lasers; bent lines: optical fiber; icons with blue edges: lenses or a beam splitter cube; QWP: quarter-wave plate; IP: image plane; IIP: intermediate image plane; BS: beam splitter; OD: optical density. Optics are shown for producing a second image on the EMCCD, but the second path was not used in this study. The gray lines denote the 808 nm beam in the focus lock apparatus.
