# Supplementary material for: Nanoscale cellular organization of viral RNA and proteins in SARS-CoV-2 replication organelles
Source: Nat Commun. 2024 May 31;15:4644. doi: 10.1038/s41467-024-48991-x (PMC11143195; doi:10.1038/s41467-024-48991-x)
Supplement: Supplementary file 3 — Reporting Summary [file 41467_2024_48991_MOESM3_ESM.pdf]

nature portfolio

Corresponding author(s): Prof. W.E. Moerner, Prof. Lei S. Qi

Last updated by author(s): 2024 April 23

Reporting Summary

Nature Portfolio wishes to improve the reproducibility of the work that we publish. This form provides structure for consistency and transparency in reporting. For further information on Nature Portfolio policies, see our Editorial Policies and the Editorial Policy Checklist.

Statistics

For all statistical analyses, confirm that the following items are present in the figure legend, table legend, main text, or Methods section.

n/a	Confirmed	
[ ]	[x]	The exact sample size (*n*) for each experimental group/condition, given as a discrete number and unit of measurement
[ ]	[x]	A statement on whether measurements were taken from distinct samples or whether the same sample was measured repeatedly
[ ]	[x]	The statistical test(s) used AND whether they are one- or two-sided<br>*Only common tests should be described solely by name; describe more complex techniques in the Methods section.*
[x]	[ ]	A description of all covariates tested
[x]	[ ]	A description of any assumptions or corrections, such as tests of normality and adjustment for multiple comparisons
[ ]	[x]	A full description of the statistical parameters including central tendency (e.g. means) or other basic estimates (e.g. regression coefficient) AND variation (e.g. standard deviation) or associated estimates of uncertainty (e.g. confidence intervals)
[ ]	[x]	For null hypothesis testing, the test statistic (e.g. *F*, *t*, *r*) with confidence intervals, effect sizes, degrees of freedom and *P* value noted<br>*Give P values as exact values whenever suitable.*
[x]	[ ]	For Bayesian analysis, information on the choice of priors and Markov chain Monte Carlo settings
[x]	[ ]	For hierarchical and complex designs, identification of the appropriate level for tests and full reporting of outcomes
[ ]	[x]	Estimates of effect sizes (e.g. Cohen's *d*, Pearson's *r*), indicating how they were calculated

Our web collection on statistics for biologists contains articles on many of the points above.

Software and code

Policy information about availability of computer code

Data collection	Andor SOLIS 4.31.30024.0; NIS Elements 4.60.
Data analysis	ImageJ 1.54f; ThunderStorm dev-2016-09-10-b1; Matlab R2023b; PYME 23.06.15; SharpViSu v2.0.3

For manuscripts utilizing custom algorithms or software that are central to the research but not yet described in published literature, software must be made available to editors and reviewers. We strongly encourage code deposition in a community repository (e.g. GitHub). See the Nature Portfolio guidelines for submitting code & software for further information.

Data

Policy information about availability of data

All manuscripts must include a data availability statement. This statement should provide the following information, where applicable:

- Accession codes, unique identifiers, or web links for publicly available datasets
- A description of any restrictions on data availability
- For clinical datasets or third party data, please ensure that the statement adheres to our policy

The data for analysis figures generated in this study are provided in the Source Data file.

Research involving human participants, their data, or biological material

Policy information about studies with human participants or human data. See also policy information about sex, gender (identity/presentation), and sexual orientation and race, ethnicity and racism.

Reporting on sex and gender	N/A
Reporting on race, ethnicity, or other socially relevant groupings	N/A
Population characteristics	N/A
Recruitment	N/A
Ethics oversight	N/A

Note that full information on the approval of the study protocol must also be provided in the manuscript.

Field-specific reporting

Please select the one below that is the best fit for your research. If you are not sure, read the appropriate sections before making your selection.

☒ Life sciences ☐ Behavioural & social sciences ☐ Ecological, evolutionary & environmental sciences

For a reference copy of the document with all sections, see nature.com/documents/nr-reporting-summary-flat.pdf

Life sciences study design

All studies must disclose on these points even when the disclosure is negative.

Sample size	Each sample preparation resulted in approximately 10 wells with different target pairs. For each prepared sample well (each label pair), typically 5-10 cells were imaged using SR microscopy and 50-100 cells were imaged using confocal microscopy. Because different targets appear in many pairs of labels, each target was imaged multiple times. The total number of infected cells imaged in SR microscopy with particular targets was: vgRNA, > 150 cells at 24 hpi, > 50 cells at 6 hpi; dsRNA, > 100 cells at 24 hpi, > 30 cells at 6 hpi; nsp12, > 40 cells at 24 hpi, > 20 cells at 6 hpi; Sec61β, > 40 cells at 24 hpi, > 25 cells at 6 hpi; nsp3, > 40 cells at 24 hpi, > 30 cells at 6 hpi; spike protein, > 40 cells at 24 hpi, > 25 cells at 6 hpi; nucleocapsid protein, > 30 cells at 24 hpi, > 20 cells at 6 hpi.
Data exclusions	No data exclusion.
Replication	The experimental measurements were replicated 3 times (4 times for vgRNA) at 24 hpi and 2 times at 6 hpi, starting with cell growth and infection at the BSL-3 facility (independent biological replicates). All biological replicates were successful.
Randomization	No randomization was used in this study. Due to the small sample randomization was not relevant for this study.
Blinding	Blinding was not possible in this study, because due to intrinsically low throughput of SR microscopy, only cells with a positive signal of SARS-CoV-2 markers were selected to be imaged and analyzed in the infected cell groups.

Reporting for specific materials, systems and methods

We require information from authors about some types of materials, experimental systems and methods used in many studies. Here, indicate whether each material, system or method listed is relevant to your study. If you are not sure if a list item applies to your research, read the appropriate section before selecting a response.

Materials & experimental systems

n/a	Involved in the study	
☐	☒	Antibodies
☐	☒	Eukaryotic cell lines
☒	☐	Palaeontology and archaeology
☒	☐	Animals and other organisms
☒	☐	Clinical data
☒	☐	Dual use research of concern
☒	☐	Plants

Methods

n/a	Involved in the study	
☒	☐	ChIP-seq
☒	☐	Flow cytometry
☒	☐	MRI-based neuroimaging

Antibodies

Antibodies used	Primary antibodies and the dilutions and concentrations used are as follows:<br>goat polyclonal anti-spike S2 (Novus Biologicals, AF10774-SP, 1:20, 10 μg/mL),<br>mouse monoclonal anti-dsRNA (J2 clone, SCICONS, 10010200, lot J2-2017, 1:200, 5 μg/mL), r<br>abbit polyclonal anti-RdRp/nsp12 (Sigma-Aldrich, SAB3501287-100UG, lot 926721011, 1:500, 2 μg/mL),<br>mouse monoclonal anti-nucleocapsid (Thermo Fisher, MA5-29981, lot XF3619701, 1:500, 2 μg/mL),<br>rabbit polyclonal anti-nsp3 (Thermo Fisher, PA5-116947, lot YA3808034B, 1:134, 5 μg/mL),<br>sheep polyclonal anti-GFP (Bio-Rad, 4745-1051, 1:1000, 5 μg/mL),<br>rabbit polyclonal anti-GFP (Novus Biologicals, NB600-308SS, lot 48142, 1:163, 5 μg/mL),<br>rabbit monoclonal anti-nsp7 (GeneTex, GTX636719, lot 44620, 1:200, 2 μg/mL),<br>mouse monoclonal anti-nsp8 (GeneTex, GTX632696, lot 42345, 1:134, 5 μg/mL),<br>mouse monoclonal anti-BrdU (MoBU-1 clone, Thermo Fisher, B35128, lot 2712999, 1:50, 2 μg/mL).<br>Secondary antibodies and the optimal dilutions and concentrations used are as follows:<br>AF647-conjugated donkey anti-mouse IgG (Thermo Fisher, A-31571, lot 2555690, 1:500, 4 μg/mL),<br>AF647-conjugated donkey anti-rabbit IgG (Thermo Fisher, A-31573, lot 2359136, 1:500, 4 μg/mL),<br>AF647-conjugated donkey anti-sheep IgG (Thermo Fisher, A-21448, lot 2454134, 1:500, 4 μg/mL),<br>CF568-conjugated donkey anti-goat IgG (Sigma-Aldrich, SAB4600074-50UL, lot 18C0723, 1:500, 4 μg/mL),<br>CF568-conjugated donkey anti-rabbit IgG (Sigma-Aldrich, SAB4600076-50UL, lot 21C1025, 1:500, 4 μg/mL),<br>CF568-conjugated donkey anti-mouse IgG (Sigma-Aldrich, SAB4600075-50UL, lot 17C1116, 1:500, 4 μg/mL),<br>CF568-conjugated donkey anti-sheep IgG (Sigma-Aldrich, SAB4600078-50UL, lot 11C1031, 1:500, 4 μg/mL),<br>CF583R-conjugated donkey anti-mouse IgG (Biotium, Custom CF Dye, lot 23C1122, 1:250, 4 μg/mL),<br>CF583R-conjugated donkey anti-rabbit IgG (Biotium, Custom CF Dye, lot 23C0811, 1:250, 4 μg/mL).
Validation	Each antibody was validated by comparing their IF signal in SARS-CoV-2 infected and non-infected cells or in cells expressing Sec61β-GFP and WT cells for the anti-GFP antibodies. The concentrations of the antibodies were optimized (Supplementary Fig. S12-13). All the antibodies are commercialized, and validation statements and references are provided by the vendor's websites:<br>https://www.novusbio.com/products/sars-cov-2-spike-s2-antibody_af10774<br>https://nordicmubio.com/products/mouse-anti-double-stranded-rna-j2/10010200<br>https://www.sigmaaldrich.com/US/en/product/sigma/sab3501287<br>https://www.thermofisher.com/antibody/product/SARS-SARS-CoV-2-Nucleocapsid-Antibody-clone-5-Monoclonal/MA5-29981<br>https://www.thermofisher.com/antibody/product/SARS-CoV-2-Nonstructural-Protein-3-Antibody-Polyclonal/PA5-116947<br>https://www.bio-rad-antibodies.com/polyclonal/green-fluorescent-protein-antibody-4745-1051.html?f=purified<br>https://www.novusbio.com/products/gfp-antibody_nb600-308<br>https://www.genetex.com/Product/Detail/SARS-CoV-2-COVID-19-nsp7-antibody-HL1301/GTX636719<br>https://www.genetex.com/Product/Detail/SARS-CoV-SARS-CoV-2-COVID-19-nsp8-antibody-5A10/GTX632696<br>https://www.thermofisher.com/antibody/product/BrdU-Antibody-clone-MoBU-1-Monoclonal/B35128<br>https://www.thermofisher.com/antibody/product/Donkey-anti-Mouse-IgG-H-L-Highly-Cross-Adsorbed-Secondary-Antibody-Polyclonal/A-31571<br>https://www.thermofisher.com/antibody/product/Donkey-anti-Rabbit-IgG-H-L-Highly-Cross-Adsorbed-Secondary-Antibody-Polyclonal/A-31573<br>https://www.thermofisher.com/antibody/product/Donkey-anti-Sheep-IgG-H-L-Cross-Adsorbed-Secondary-Antibody-Polyclonal/A-21448<br>https://www.sigmaaldrich.com/US/en/product/sigma/sab4600074<br>https://www.sigmaaldrich.com/US/en/product/sigma/sab4600076<br>https://www.sigmaaldrich.com/US/en/product/sigma/sab4600075<br>https://www.sigmaaldrich.com/US/en/product/sigma/sab4600078

Eukaryotic cell lines

Policy information about cell lines and Sex and Gender in Research

Cell line source(s)	HEK 293T cells (ATCC, CRL-3216);<br>Vero E6 cells (ATCC, CRL-1586);<br>Vero E6-TMPRSS2 cells.
Authentication	Cell lines were authenticated by the supplier ATCC. We did not perform any additional authentication upon reception. We made bulk stocks for each cell line after recovering from the original frozen vials. We discard the cells after passage for 30 days and thaw new cells from liquid nitrogen stocks. Cell morphology was monitored at each passage by microscope.
Mycoplasma contamination	Cells were routinely tested (PCR based test) and were mycoplasma negative.
Commonly misidentified lines<br>(See ICLAC register)	No commonly misidentified cell lines were used in this manuscript.

Plants

Seed stocks	N/A
Novel plant genotypes	N/A
Authentication	N/A
